# Scanning Electron Microscopic Evaluation of Residual Smear Layer Following Preparation of Curved Root Canals Using Hand Instrumentation or Two Engine-Driven Systems

**DOI:** 10.7508/iej.2015.04.005

**Published:** 2015

**Authors:** Abbasali Khademi, Masoud Saatchi, Mohammad Mehdi Shokouhi, Badri Baghaei

**Affiliations:** a *Torabinejad Dental Research Center, Department of Endodontics, Dental School, Isfahan University of Medical Sciences, Isfahan, Iran; *; b * Department of Endodontics, Dental School, Shiraz University of Medical Sciences, Shiraz, Iran****; ***; c *Department of Endodontics, Dental School, Rafsanjan University of Medical Sciences, Rafsanjan, Iran*

**Keywords:** Canal Preparation, Endodontics, Irrigants, Scanning Electron Microscopy, Smear layer

## Abstract

**Introduction:**

In this experimental study, the amount of smear layer (SL) remnants in curved root canals after chemomechanical instrumentation with two engine-driven systems or hand instrumentation was evaluated.

**Methods and Materials:**

Forty-eight mesiobuccal roots of mandibular first molars with curvatures ranging between 25 and 35 degrees (according to Schneider’s method) were divided into three groups (*n*=16) which were prepared by either the ProTaper Universal file series, Reciproc single file system or hand instrumentation. The canals were intermittently irrigated with 5.25% NaOCl and 17% (ethylenediaminetetraacetic acid) EDTA, followed by distilled water as the final rinse. The roots were split longitudinally and the apical third of the specimens were evaluated under 2500× magnification with a scanning electron microscope (SEM). The mean scores of the SL were calculated and analyzed using the non-parametric Kruskal-Wallis and Mann-Whitney U tests.

**Results:**

The mean scores of the SL were 2.00±0.73, 1.94±0.68 and 1.44±0.63 µm for the ProTaper Universal, Reciproc and hand instrumentation, respectively. Mean score of SL was significantly less in the hand instrumentation group than the ProTaper (*P*=0.027) and Reciproc (*P*=0.035) groups. The difference between the two engine-driven systems, however, was not significant (*P*=0.803).

**Conclusion:**

The amount of smear layer in the apical third of curved root canals prepared with both engine-driven systems was similar and greater than the hand instrumentation technique. Complete cleanliness was not attained.

## Introduction

Cleaning and shaping of the root canal system is critical for the success of root canal therapy [1]. During physical instrumentation, accumulation of organic pulpal materials and inorganic dentinal debris produces an irregular matter known as smear layer (SL) [2]. Some investigators are of the opinion that the SL prevents disinfectants [3] and sealers [4] from penetrating into the dentinal tubules. It can also compromise the sealing ability of root filling materials [5]. However, the issue of the SL removal remain controversial [6]. 

The use of nickel-titanium (NiTi) engine-driven instruments has progressively increased among dentists [7]. ProTaper (PTR) (Dentsply Maillefer, Ballaigues, Switzerland) is one of the most commonly used and pioneer rotary systems, which is safe and effective for preparation of curved root canals [8]. This system incorporated sequential use of at least five instruments. Reciproc (RCP) (VDW, Munich, Germany) is a single-file engine-driven system and the manufacturer claims that the whole steps of canal preparation can be carried out using a single file [9]. This file is made of the M-Wire NiTi alloy the advantages of which are the increased cyclic fatigue resistance as well as flexibility [9]. The reciprocating 30^°^ clockwise and 150^°^ counterclockwise movements of RCP files play an important role in decreasing the rate of cyclic fatigue fracture [10]. Reciprocating files can also reduce the bacterial count during root canal preparation [11]. However, like any other instrumentation procedure, they produce SL, which covers the dentinal walls and occludes the dentinal tubules [12, 13].

**Figure 1.**
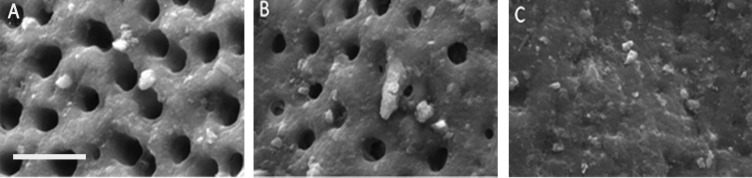
*A)* A scanning electron microscopic (SEM) image of a specimen with *score I* of smear layer; the regular pattern of open dentinal tubules without the presence of the smear layer; *B)* SEM image of a specimen with *score II*; some dentinal tubules are patent whereas the rest of the surface is covered with the smear layer; *C)* SEM image of a specimen with *score III*; the canal wall is totally covered with the smear layer whereas no patent dentinal tubules can be seen (original magnification 2500×, bar=10 μm)

The apical third of the root canal system is the most difficult area to clean due to the complex anatomy and presence of deltas, lateral canals, isthmi and ramifications [14]. The aim of this experimental* in vitro* study was to compare the SL remaining in the apical region of curved root canal walls after chemomechanical preparation using PTR, RCP and traditional hand instrumentation (HI) by means of scanning electron microscopy (SEM).

## Materials and Methods

The Ethics Committee of Isfahan University of Medical Sciences approved the protocol of the study. Forty-eight first mandibular molars from adult subjects, with 25^°^ to 35^°^ curvature of the mesial root canals according to the Schneider’s method [15], were selected. The teeth were kept in deionized water containing few amounts of thymol crystals. The access cavities were prepared and the mesiobuccal canals were negotiated. To estimate the working length, a #10 K-file (Mani, Tochigi, Japan) was inserted within the mesiobuccal canal until the tip of the file was visible through the apical foramen and then 0.5 mm was subtracted from this measurement. 

The specimens were randomly divided into three groups (*n*=16). In the PTR group, the canals were prepared by shaping (SX, S1 and S2) and finishing (F1, F2, and F3) files with a crown-down approach and continuous “in-and-out” movements for F files and “brushing” movements for S files, as recommended by the manufacturer. 

In the RCP group, the canals were prepared with the R25 (25/0.08) file installed on a gear reduction handpiece (Sirona Dental Systems GmbH, Bensheim, Germany) powered by a torque-controlled motor (Silver; VDW GmbH, Munich, Germany) that was set on ‘‘Reciproc All’’ mode with “in-and-out” pecking movements. 

In the hand instrumentation group, the canals were prepared by the step-back technique; coronal flaring was accomplished using sizes 1 to 3 Gates Glidden drills (Dentsply Maillefer, Ballaigues, Switzerland). Then #15-30 Flexofile instruments (Dentsply Maillefer, Ballaigues, Switzerland) were used with “watch-winding” motion. After the #30 Flexofile reached the working length (WL) without any resistance, the apical third of the canal was considered fully prepared. Then the preparation of the middle and coronal thirds was accomplished by #35-60 Flexofile instruments. Successive larger files were used with 1 mm-reducing increments from the previous file. In all the groups, the apical patency was maintained using a #10 K-file. The flutes were cleaned after using each instrument or every three pecking movements in the RCP group. 

After using each instrument in PTR group or after three pecking movements in the RCP group, irrigation was carried out by 2 mL of 5.25% NaOCl, followed by 2 mL of 17% ethylenediaminetetraacetic acid (EDTA) followed by final rinse with 3 mL of distilled water. Disposable syringes with 30-gauge NaviTip needles (Ultradent, South Jordan, UT, USA) were used for irrigation. The needle was inserted 2-3 mm shorter than the WL and progressively within 1 mm from the WL without binding. The needle was then pulled back 1 mm, and irrigation solution was injected into the canal. The canals were then dried using paper points. The access cavities were sealed with Cavit (ESPE-Premier, Norristown, PA, USA) and the specimens were coded for blinded assessment.

Preparation of the samples for SEM started with preparing two longitudinal grooves on the buccal and lingual surfaces using a #1 diamond disk (D&Z, Diamant, Germany). The roots were then split vertically with a chisel. The apical thirds of the canals were evaluated by SEM (Model LEO-1400, England) under 2500× magnification. The SEM images were scored according to the scoring system recommended by Zmener [16]: *Score I*; Regular open dentinal tubules with no layers, *score II;* Some tubules are open while others are occluded with the SL and *score III*; All the tubules are obscured by the SL covering the canal walls. 

Two blinded examiners evaluated the samples and a third examiner was asked to render an independent judgment if evaluations of the two investigators did not match. The mean score of each group was calculated. Data were then analyzed using SPSS software version 16 (SPSS Inc, Chicago, IL, USA). Statistical analysis was carried out by the Kruskal-Wallis and Mann-Whitney U tests. Inter-rater agreement was evaluated using the kappa coefficient. The level of significance was set at 0.05.

## Results

The inter-rater agreement between the two investigators was 0.92, indicating a reliable scoring. Figure 1 shows the SEM photomicrographs of the SL for each score. The distribution of SL scoring for each group is presented in Table 1. There was no significant difference between the PTR and the RCP groups (*P*=0.803). However, there were statistically significant differences between the HI group and both the PTR (*P*=0.027) and RCP (*P*=0.035) groups.

## Discussion

In this study the remaining SL in the curved root canals after preparation with the RCP and PTR systems and HI was investigated using SEM. We showed that both RCP and PTR systems produced more SL than HI technique. However, the remaining SL in both engine-driven systems was similar. 

The SL has a thickness of 1-2 μm and is produced during the cleaning and shaping of the root canal system [4]. Some investigators have supported the persistence of the SL because it may block the dentinal tubules and restrict bacterial or toxin penetration by changing the dentinal permeability [17, 18]. Others have supported the removal of the SL because it permits diffusion and penetration of irrigants, sealers and medications into the dentinal tubules [6, 19] and increases the push-out bond strength of sealers and biomaterials to the root canal dentin [20-22]. The results of a systematic review and meta-analysis supported the removal of the SL [6]. Moreover, the presence of the SL might be considered an indicator of cleaning ability of root canal preparation techniques and instruments. 

Some studies have used the 3-score index recommended by Zmener *et al.* [16] for scoring of the SL remaining on the root canal wall [23, 24]. Others used a 5-score index recommended by Hülsmann *et al.* [25, 26]. Both of them are acceptable enough and have been used in different studies. However, in this study we used a 3-score index recommended by Zmener *et al.* [16]. Different magnifications of SEM have been used for scoring of the SL remaining on the root canal walls [27, 28]. Also the 2500× magnification enabled seeing more details for easier detection of the SL and patent dentinal tubules, leading to more convenient scoring. 

**Table 1:** N (%) of SL scoring with SEM (PTR**=**ProTaper, RCP=Reciproc and HI=hand instrumentation

**Group (N)**	**Score ** ***I*** ** N(%)**	**Score ** ***II*** ** N(%)**	**Score ** ***III*** ** N(%)**
**PTR (16)**	4 (25)	8 (50)	4 (25)
**RCP (16)**	10 (63)	5 (31)	1 (6)
**HI (16)**	4 (25)	9 (56)	3 (19)
**Total (48)**	18 (38)	22 (46)	8 (17)

Peters *et al.* [29] showed that while there is no difference between the amount of remaining debris and the SL after using LightSpeed instruments and ProFile in canals irrigated with distilled water, there was a significant difference regarding this cleanliness after using NaOCl and EDTA. Therefore, in the present study 5.25% NaOCl and 17% EDTA were used and the final flushing was done with distilled water in order to simulate the clinical conditions.

None of the root canal preparation systems was able to completely remove the SL, which is compatible with the literature [14]. The HI specimens had the least remaining SL compared to RCP and PTR, as reported by Arya *et al*. [30]. Lumley *et al.* [31] claimed that instruments with smaller tapers, clean canals more efficiently. The coronal segment of more tapered file binds on the canal walls, while the apical section of the file is passively inserted in the canal. Moreover, applying lateral pressure with NiTi instruments may lead to production of more SL. On the other hand, stainless steel instruments are stiffer, and therefore the lateral pressure is feasible with them [32]. The increased number of apical rotations in rotary instruments can be another reason for producing more SL [30].

Poggio *et al.* [13] showed that RCP system produced more SL than the Mtwo files in the apical third of the canal. They claimed that conventional continuous rotary NiTi instruments seem to be better for obtaining clean dentinal canal walls. In the present study, there were no significant differences in the SL scores between the PTR and the RCP groups. However, the PTR group scores were slightly more than those of RCP group, which is consistent with the results reported by Burklein* et al*. [33]. It might be attributed to the different design of these two systems. The RCP file has a S-shaped cross-section with sharp cutting edges while the PTR file has a triangular cross-section and three cutting edges with a small flute. A smaller flute of the instrument leads to less SL removal [33].

## Conclusion

Based on the findings of this study, the engine-driven systems produce more smear layer than the traditional hand instruments. The smear layer remaining in the apical third of curved root canals was similar with both systems. Complete cleanliness of the canal walls was not attained.
